# Self-Supported Co_3_O_4_@Mo-Co_3_O_4_ Needle-like Nanosheet Heterostructured Architectures of Battery-Type Electrodes for High-Performance Asymmetric Supercapacitors

**DOI:** 10.3390/nano12142330

**Published:** 2022-07-07

**Authors:** Yedluri Anil Kumar, Himadri Tanaya Das, Phaneendra Reddy Guddeti, Ramesh Reddy Nallapureddy, Mohan Reddy Pallavolu, Salem Alzahmi, Ihab M. Obaidat

**Affiliations:** 1 Department of Physics, United Arab Emirates University, Al Ain 15551, United Arab Emirates; yedluri.anil@gmail.com; 2 National Water and Energy Center, United Arab Emirates University, Al Ain 15551, United Arab Emirates; s.alzahmi@uaeu.ac.ae; 3 Centre of Advanced Materials and Applications, Utkal University, Vanivihar, Bhubaneswar 751004, India; himadridas@utkaluniversity.ac.in; 4 Department of Physics, Sri Venkateswara Vedic University, Tirupati 517502, India; phaneendra369@gmail.com; 5 School of Chemical Engineering, Yeungnam University, Gyeongsan 38541, Korea; rameshsun999@gmail.com; 6 Department of Chemical & Petroleum Engineering, United Arab Emirates University, Al Ain 15551, United Arab Emirates

**Keywords:** Co_3_O_4_@Mo-Co_3_O_4_ nanocomposite, binder free electrode, supercapacitor, hydrothermal, energy storage

## Abstract

Herein, this report uses Co_3_O_4_ nanoneedles to decorate Mo-Co_3_O_4_ nanosheets over Ni foam, which were fabricated by the hydrothermal route, in order to create a supercapacitor material which is compared with its counterparts. The surface morphology of the developed material was investigated through scanning electron microscopy and the structural properties were evaluated using XRD. The charging storage activities of the electrode materials were evaluated mainly by cyclic voltammetry and galvanostatic charge-discharge investigations. In comparison to binary metal oxides, the specific capacities for the composite Co_3_O_4_@Mo-Co_3_O_4_ nanosheets and Co_3_O_4_ nano-needles were calculated to be 814, and 615 C g^−1^ at a current density of 1 A g^−1^, respectively. The electrode of the composite Co_3_O_4_@Mo-Co_3_O_4_ nanosheets displayed superior stability during 4000 cycles, with a capacity of around 90%. The asymmetric Co_3_O_4_@Mo-Co_3_O_4_//AC device achieved a maximum specific energy of 51.35 Wh Kg^−1^ and power density of 790 W kg^−1^. The Co_3_O_4_@Mo-Co_3_O_4_//AC device capacity decreased by only 12.1% after 4000 long GCD cycles, which is considerably higher than that of similar electrodes. All these results reveal that the Co_3_O_4_@Mo-Co_3_O_4_ nanocomposite is a very promising electrode material and a stabled supercapacitor.

## 1. Introduction

With broad applicability in the electronics, power systems, communication systems, and automobile sectors, supercapacitors have been recognized as being highly reliable among the numerous forms of energy storage devices. Supercapacitors exhibit high power densities and high charge storage abilities with long-term stability compared to batteries [1]. The cost-effective and less hazardous properties of supercapacitor components have motivated researchers to explore different electrode materials. Still, the practical usability of supercapacitors is limited due to their low energy densities for which battery-type electrodes are currently preferred. A wide range of transition mono-/multi-metallic oxides or hydroxides has been investigated as electrode materials for supercapacitors [2,3]. Among these, cobalt oxides have been found to possess high theoretical specific capacities [4]. The electrochemical properties of cobalt oxides can be improved by different strategies like tuning their morphology, doping with other metals, creating oxygen vacancies, etc. The tuning morphology is governed by the synthesis process, temperature, concentration, and reaction time. Among the various synthesis processes, the hydrothermal method is an old technique for synthesizing various nanomaterials. Interestingly, nanomaterials derived from hydrothermal methods with different shapes and sizes, dopants, core-shell, heterostructures, etc. have been found to be efficient for energy storage applications [5,6].

It has been reported that CoMoO_4_ hexagonal 2D-nanosheets have high charge storage capabilities [7]. Such bimetallic oxides also have the advantage of the synergetic effect of both metals, where either both metals take part in redox reactions or one of them supports the electrochemical reaction of the other. Interestingly, oxygen vacancies are created in CoMoO_4-x_ by hydrogenation of the hydrothermally obtained CoMoO_4_ [8]. A theoretical study discovered that the oxygen vacancies have improved carrier density, thus increasing the electronic conductivity. The oxygen vacancy also acts as an active site for OH^-^ intake and accelerates the charge transfer kinetics. Another strategy, i.e., doping the parent nanomaterial, has been widely explored due to the cost-effective approach to synthesis and ease of tuning the properties which it offers. For example, rare-earth metal (La, Nd, Gd, Sm) doping of Co_3_O_4_ synthesized by the polymer combustion method was employed for the study of the transfer of ions and electrons in supercapacitors [9]. Mo-doping of Co_3_O_4_ by various methods (sol-gel, electrodeposition, and hydrothermal) has been investigated as a means to synthesize potential electrode materials [10,11]. Shen et al. developed Mo-Co_3_O_4_ nanosheets as a battery-type electrode for supercapacitor applications and reported a specific capacity of 128.2 mAh/g at 1 A/g with 95% of capacity retention after long-term cyclings [12]. The experimental results were substantiated by theoretical calculations; it was shown that Mo-doping of Co_3_O_4_ modifies the bandgap and increases the electronic conductivity of the parent material, which enhances charge storage capability.

Recently, heterostructures have been designed as electrode materials for supercapacitors due to their extraordinary electrochemical performance and self-supported nanostructures with high structural and chemical stability [13]. A core-shell of hierarchical 3D NiCo_2_O_4_@ZnWO_4_ was synthesized by the hydrothermal method for an all-solid-state symmetric supercapacitor (SSC) [14]. A multicomponent MnMoO_4_/MnCO_3_ hybrid synthesized by a one-step hydrothermal method with urea as the reaction fuel was found to store ample charge with good reversibility [15]. Zhao et al. engineered Fe_2_O_3_ nanoneedle arrays with typical mesoporous structures and NiCo_2_O_4_/Ni(OH)_2_ hybrid nanosheet arrays on SiC nanowire skeletons for asymmetric supercapacitors [16]. Binary heterojunction nanocomposites of CoTiO_3_@Co_3_O_4_/N-single bond CNO matrix were established with excellent electrochemical properties [17]. Similarly, a hybrid, interconnected structure of nanoplates and nanowires of NiCo_2_O_4_ was found to have large surface area to support redox reactions [18]. A nanocomposite of needle-like Co_3_O_4_ and graphitic N-CNOs nanosheets was synthesized by a simple solvothermal and pyrolysis method [19]; it showed very high specific capacitance and good rate capability. Further, the asymmetric device with activated carbon (AC) showed good energy density and Coulombic efficiency. Such work proves that needle-like nanostructures along with nanosheets are appropriate electrodes for supercapacitors. There are many advantages of using nanomaterials, such as: (i) large surface area; (ii) synergistic charge storage; (iii) more electroactive sites exposed to the electrolyte; and (iv) time and cost-saving production.

In this work, a Co_3_O_4_@Mo-Co_3_O_4_ nanosheet composite heterostructure was synthesized by hydrothermal and pyrolysis method. As a result of the synergistic redox features between the Co_3_O_4_ nano-needles and Mo-Co_3_O_4_ nanosheets, the Co_3_O_4_@Mo-Co_3_O_4_ electrode displayed superior electrochemical activities with a specific capacity of 814 C g^−1^ (1 A g^−1^) and retention of 90% after 4000 long-cycles. In addition, an asymmetric supercapacitor (ASC) with continuous cycling stability was assembled utilizing Co_3_O_4_@Mo-Co_3_O_4_ and activated carbon (AC). The fabricated Co_3_O_4_@Mo-Co_3_O_4_//AC ASC displayed great electrochemical capacity, stability, and superior conductivity.

## 2. Experiment Procedure

### 2.1. Chemical Details

All chemicals were purchased from Sigma Aldrich and used without further processing. Cobalt nitrate hexahydrate (Co(NO_3_)_2_.6H_2_O), sodium molybdate (NaMoO_4_), and urea were used to synthesize the electrode material. N-Methyl-2-pyrrolidone (NMP, analytical grade), polyvinylidene fluoride (PVDF, analytical grade), nickel (Ni) foam, and activated carbon (AC, analytical grade) were used for electrode fabrication.

### 2.2. Synthesis of Co_3_O_4_ and Co_3_O_4_@Mo-Co_3_O_4_

**Figure 1:**
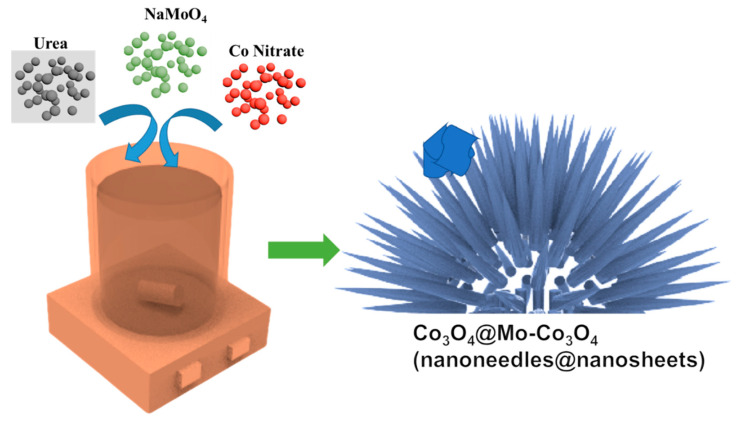
Schematic of Co_3_O_4_@Mo-Co_3_O_4_ needle-like nanosheets, synthesized by hydrothermal method.

The hetero-structure nanomaterial was synthesized via a facile hydrothermal method followed by low-temperature calcination. Pure precursors, i.e., cobalt nitrate, sodium molybdate, and urea, were used as-purchased. First, 2 mmol of Co(NO_3_)_2_.6H_2_O (0.582 g), 1 mMol NaMoO_4_ (0.206 g), and 10 mM urea (0.6 g) were completely dissolved in distilled water (25 mL) and ethanol (10 mL). Then, the 35-mL solution was transferred to a Teflon-lined autoclave for hydrothermal synthesis. The autoclave was subjected to heating to 120 °C and maintained for 10 h. Then, the autoclave was cooled to room temperature. The hydrothermal sample was washed with distilled water and ethanol to remove unreacted precursors or impurities, followed by drying at 60 °C. Finally, the obtained powder was calcinated at 350 °C for 3 h at a heating rate of 5 °C/min. Figure 1 presents a schematic view of the synthesis of the heterostructured Co_3_O_4_ nanoneedles and Mo-Co_3_O_4_ nano-petals. The low temperature used in the hydrothermal treatment allowed nucleation and the growth of nanoneedles and nanosheets to occur, while the post-annealing improved the crystallinity of the synthesized oxides.

### 2.3. Characterizations

Detailed characterizations and an electrochemical analysis are available in the supporting file. In addition, the device fabrication procedure and calculation method of the specific capacity, energy, and power densities are provided and applied to the methods and calculations reported in previous literature [15,17,19,20,21].

### 2.4. Asymmetric Supercapacitor Preparation

For a three-electrode configuration, Co_3_O_4_ and Mo-Co_3_O_4_ coated on Ni foam were utilized as a working electrode, platinum wire as a counter electrode, and Ag/AgCl as a reference electrode, with 2.0 M KOH as the active electrolyte. For a two-electrode setup, Mo-Co_3_O_4_ coated on Ni foam was utilized as a positive electrode and activated carbon (AC) as a negative electrode, while Whatman filter paper— dipped in 2 M KOH solution and then placed between the positive and negative electrodes—was used as a separator. The mass-loading in the three-electrode setup was around 3 mg; in the two-electrode setup, an active mass of 8.7 mg was loaded onto the positive electrode (M) and 1 mg was loaded onto the negative electrode (AC). These values were chosen to balance the charge on both electrodes. The CV measurements for the ASC device were carried out in the potential range of −0.1 to 0.6 V at various scanning rates from 10 to 30 mV s^−1^, whereas GCD characterizations were performed in potential ranges from 0 to 1.6 V at various current densities, i.e., ranging from 1 to 10 A g^−1^. Moreover, EIS studies for both the two and three-electrode setups were carried out with an open circuit potential (OCP) ranging from 1 Hz to 100 kHz, with an AC perturbation of 5 mV.

In a three-electrode setup, the specific capacities were calculated from the GCD curves using the below equation:

(1) $$C=\frac{I\cdot \;\Delta t}{m\;}$$

where *C* is the specific capacity (C/g), *I* indicates the discharge current (A), Δ*t* is the discharge time (s), and *m* represents the mass of the active material (g). In the two-electrode setup, the specific capacitance (measured from GCD plots) of the active electrodes was calculated from the following equation:

(2) $$C_{s}=\frac{I\cdot \;\Delta t}{m\cdot \;\Delta V}$$

To assemble the supercapacitor device, the charge stored on the positive and negative electrodes was described by *q*^+^ = *q*^−^. The mass balance of the positive and negative electrodes was estimated by:

(3) $$\frac{m_{+}}{m_{-}}=\frac{C_{s-}x\Delta V_{-}}{C_{s+}x\Delta V_{+}}$$

Also, in the two-electrode setup, the energy density (Wh/Kg) and power density (W/Kg) were measured as follows:

(4) $$E=\frac{1}{2}C_{s}{\left(\Delta V\right)}^{2}$$

(5) $$P=\frac{E}{\Delta t}$$

where *C_s_* is the specific capacitances (F/g), Δ*t* is the discharge time (s), Δ*V* is the operating voltage window (V), *m* is the active mass of the working material (g), *E* is the energy density (Wh/kg), and *P* is the power density (W/kg).

## 3. Results and Discussion

**Figure 2:**
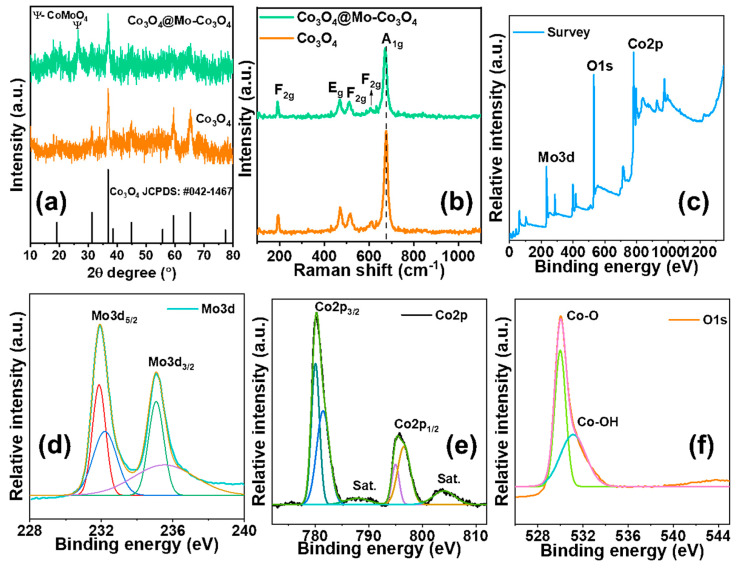
(**a**,**b**) XRD pattern and Raman spectra of Co_3_O_4_ and Co_3_O_4_@Mo-Co_3_O_4_ needle-like nanosheet heterostructure, (**c**) XPS survey spectra of Co_3_O_4_@Mo-Co_3_O_4_, (**d**) deconvoluted high-resolution spectra of Mo3d, (**e**) deconvoluted high-resolution spectra of Co2p, and (**f**) deconvoluted high-resolution spectra of O1s.

The X-ray diffraction patterns of Co_3_O_4_ and Mo-Co_3_O_4_ were studied in the 2θ range of 10–80°, as presented in Figure 2a. The diffraction patterns demonstrated that the prepared heterostructure was polycrystalline, presenting several peaks which were consistent with Co_3_O_4_ planes. The diffraction patterns of the Co_3_O_4_ and Mo-Co_3_O_4_ heterostructure showed peaks at the (220), (311), (400), (511), and (440) planes, which corresponded to the cubic crystal structure of the Co_3_O_4_ phase. All of the planes matched with the JCPDS card no: 042-1467 [19]. The diffraction pattern showed no peaks corresponding to cobalt or molybdenum, indicating that no extra phases existed in the heterostructure. The XRD pattern of the Mo-doped Co_3_O_4_ heterostructure exhibited an additional peak at the (002) plane. Irrespective of the (002) plane, all the remaining planes showed pure Co_3_O_4_, indicating that no structural deformation had occurred in the host Co_3_O_4_ lattice upon Mo-doping. This demonstrates that Mo ions were successfully substituted in the Co lattice positions in the Co_3_O_4_ matrix.

Rietveld refinement was also used to determine the presence of Mo in the Co_3_O_4_ lattice for the Co_3_O_4_@Mo-Co_3_O_4_ heterostructure. In this analysis, R weighted profile (R_wp_), R profile (R_p_), R structure factor, R Bragg factor (R_Bragg_), and goodness of fit (GOF) structural parameters were evaluated (as shown in Figure S1) using the EXPO software. The unit cell parameters were also calculated using the Rietveld refinement results. Table 1 shows the estimated Rietveld refinement parameters, including R_p_, R_wp_, GOF, R structure factor, R_Bragg_, and the unit cell parameters. The quality of Mo substitution in the Co_3_O_4_ matrix can be determined from the GOF value, which is 0.805.

Table 2 displays the site occupancy values of ions distributed with the corresponding fractional coordinate values. Co_3_O_4_ contains three Co sites (Co1, Co2, Co3) and two O sites (O1 and O2). The unit cell lattice parameters are included in Table 1. Further, the crystal structure and atomic bonding in Co_3_O_4_ were evaluated using the Vesta software. Co_3_O_4_ demonstrated a cubic crystal structure with space group Fd-3m (space group number: 227), with 130 atoms distributed with 168 bonds. Table 2 lists the structural parameters, while Figure 2 depicts the crystal structure.

Figure 2b shows Raman spectra of the Co_3_O_4_ and Co_3_O_4_@Mo-Co_3_O_4_ heterostructure. The Raman spectrum of intense peaks showed the Mo-O-Co stretching vibrations for Co_3_O_4_@Mo-Co_3_O_4_. The intense peaks in both samples may have arisen due to the vibrations of Co-O bonds representing the breathing mode of point phonons in the A_1g_ symmetry [5]. Several small peaks appeared due to the scattering of the F_2g_ or E_g_ phonons [7]. These bands can be integrated into Mo-O-Mo, Mo-O, and MoO_4_ vibrations [8]. The results suggest that the synthesis of the Co_3_O_4_@Mo-Co_3_O_4_ heterostructure composite was successful.

In addition, FTIR analysis was performed on the Co_3_O_4_@Mo-Co_3_O_4_ needle-like nanosheet heterostructure for additional phase evolution, as presented in Figure S2. This is the first reported FTIR analysis of Mo-doped Co_3_O_4_ in a supercapacitor-related publication. The sharp peaks at 560 and 665 cm^−1^ correspond to pure Co_3_O_4_, comprising Co-O stretching vibrations [22]. These two bands confirm the presence of Co_3_O_4_, which is related to the OB3 vibration (B = Co^3+^ in an octahedral hole); the other band is related to the ABO_3_ vibration (A = Co^2+^ in a tetrahedra hole) [23]. The small peaks at around 3425 and 1645 cm^−1^ resulted from the O-H groups [24]. The shift could be observed for the Mo-doped Co_3_O_4_, and was attributed to a change in the surface area as well as a surface defect due to the doping [25]. The peak at 1058 cm^−1^ belonged to the carbonate groups which resulted from air contamination during the reaction of oxide with Co_2_ [26].

The chemical composition and valance state of the Co_3_O_4_@Mo-Co_3_O_4_ needle-like nanosheet heterostructure were analyzed using X-ray photoelectron spectroscopy (XPS), revealing the oxidization state of the transition metal ion. Figure 2c displays the spectra of Mo-Co_3_O_4_, showing Mo, Co, and O peaks in addition to C. The core-level photoelectron spectra of Mo3d, Co2p, and O1s are presented in Figure 2d–f. The Mo3d core transition was considered by a doublet due to the spin-orbit coupling resulting from the 3d_5/2_ and 3d_3/2_ components. The binding energies of Mo3d_5/2_ and Mo3d_3/2_ were 231.92 ± 0.1 eV and 235.06 ± 0.1 eV, respectively. The local structure of the Mo atoms in the Co_3_O_4_ lattice offered information about its chemical state and was primarily responsible for these peak placements. The detected binding energy values of Mo differed from those of Mo-O-related compounds, indicating that the Mo cations had been perfectly substituted in the Co sites of the cubic structure.

Figure 2c shows two distinct peaks at 780.15 ± 0.1 eV and 795.64 ± 0.1 eV, which belong to 2p_3/2_ and 2p_1/2_, respectively. The energy difference between the Co 2p_3/2_ and 2p_1/2_ splitting was 15.49 eV, which indicated the existence of Co^2+^ and corresponded to the existence of Co_3_O_4_. It can be noted that the binding energy difference ΔE between the Co2p_1/2_ and Co2p_3/2_ (15 eV) observed in the complex was equal to that found for Co(III), which was in agreement with the reported value [27]. The peak detected at 530.26 ± 0.1 eV was attributable to O1s linked to Co and Mo atoms in the lattice oxygen (see Figure 2d). Because the activation energy for oxygen diffusion is substantially higher than that for interstitial Co atoms and Mo^6+^ ions, enough oxygen atoms from the atmosphere can diffuse into the Co_3_O_4_ lattice to fill up the new oxygen vacancies created by the increase of Co(III) and Mo^6+^ ions during Mo:Co_3_O_4_ development. The existence of Co and O in XPS spectra is consistent with the XRD patterns.

**Figure 3:**
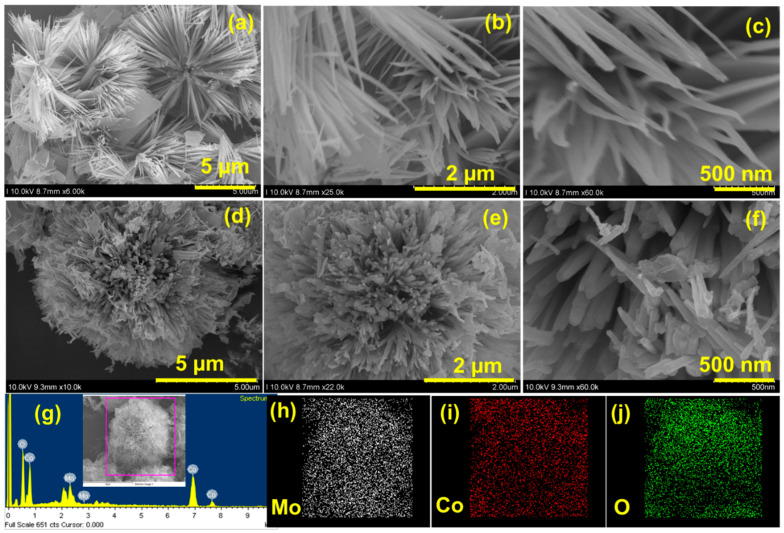
(**a**–**f**) FE-SEM images of Co_3_O_4_@Mo-Co_3_O_4_ needle-like nanosheet heterostructure at different magnifications, (**g**) EDS spectra: inset mapping image of Co_3_O_4_@Mo-Co_3_O_4_, (**h**) elemental map of Mo, (**i**) elemental map of Co, and (**j**) elemental map of O.

The morphology of Co_3_O_4_@Mo-Co_3_O_4_ was characterized by FESEM, as shown in Figure 3. The one-pot hydrothermal synthesized Co_3_O_4_@Mo-Co_3_O_4_ was found to be heterostructured. It is worth mentioning that the heterostructures composed of the monolayer Co_3_O_4_ and some Mo-Co_3_O_4_ nanosheets demonstrated eminent structural formation. The images in Figure 3a–f show the Co_3_O_4_@Mo-Co_3_O_4_ at different magnifications. A bundle of nanoneedles of Co_3_O_4_ was arranged over a bud to give a flower-like nanostructure, while the Mo-Co_3_O_4_ nanosheets were stuck on/in the nanoneedles. The fact that the self-supported heterostructure formed without the addition of any surfactant or template is quite interesting. The Co_3_O_4_ and Mo-Co_3_O_4_ heterostructures exhibited excellent metallic characteristics and great structural stability, delivering better electronic conductivity and structural integrity than pristine composites. Moreover, self-assembled particles reduce the surface energy, enhancing the stability of the structure. Within the hierarchical heterostructures, both core and shell are active materials, and the core-shell heterostructures enable easy access to electrolytes. Therefore, both of them can effectively contribute to the capacity. Figure 3g shows the EDS spectra obtained for the elemental studies, whereas Figure 3h–j shows the elemental mapping for the Mo, Co, and O present in the Co_3_O_4_@Mo-Co_3_O_4_ needle-like nanosheet heterostructure. It is expected that the architecture can be beneficial for boosting electrochemical performance.

**Figure 4:**
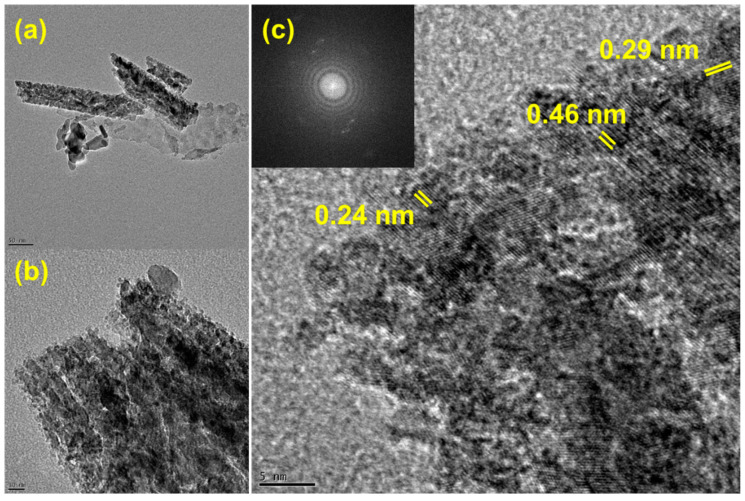
(**a**,**b**) TEM images at different magnifications, (**c**) high-resolution TEM image of Co_3_O_4_@Mo-Co_3_O_4_ needle-like nanosheet heterostructure: inset SAED pattern.

HRTEM images of the Co_3_O_4_@Mo-Co_3_O_4_ heterostructure are depicted in Figure 4, where the resolutions in a, b, and c are 50, 10, and 5 nm, respectively. Figure 4a,b clearly convey that there are different shapes of nanoneedles, i.e., a flower and small sheets attached to it. It was observed that the wide exposed nanoneedles in flower form provided space for the small sheets inside them, as well as sharp tips to hold nanosheets. Thus, the hydrothermal growth of the heterostructured Co_3_O_4_ nanoneedles and Mo-Co_3_O_4_ nanoplates resulted in the creation of interesting nanostructured electrode materials. The highest resolution image (Figure 4c) shows fringes, where the SAED pattern (inset in Figure 4c) confirms the highly crystalline nature of the hetero-structured nanomaterials. The precise spacings of 0.24, 0.29, and 0.46 nm were well-matched with the (311), (220), and (111) planes of Mo-Co_3_O_4_. The well-organized rings and dots seen in the SAED pattern prove the crystallinity nature of the electrode materials that could be beneficial in structure retention for long-term stability. There is no doubt that such an architecture would support efficient electrochemical performance in supercapacitor applications.

### 3.1. Electrochemical Properties of Electrode Materials

**Figure 5:**
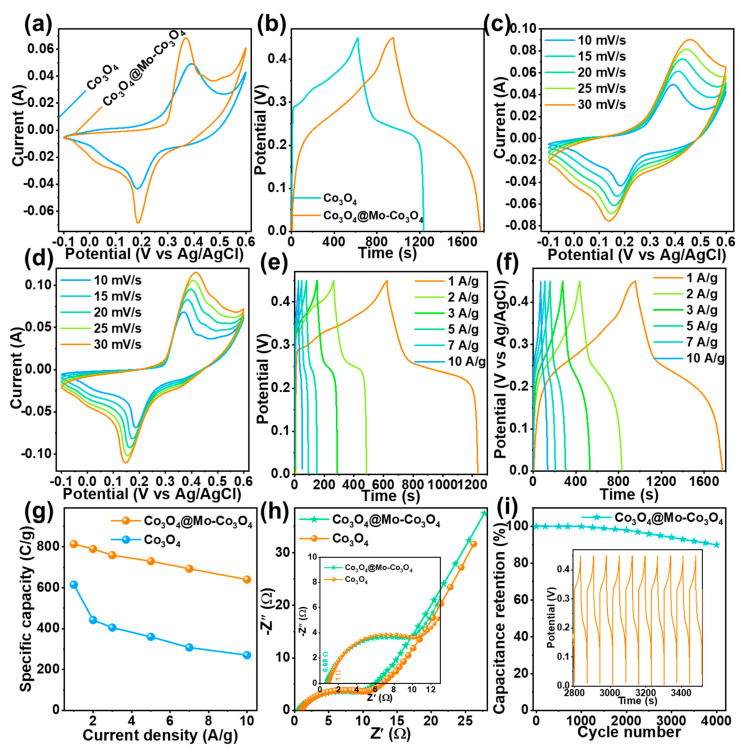
(**a**,**b**) Comparison plots of CV at 10 mV/s scan rate and GCD at 1 A/g current density for Co_3_O_4_ and the Co_3_O_4_@Mo-Co_3_O_4_ heterostructure, (**c**,**d**) CV curves at scan rates of 10–30 mV/s, (**e**,**f**) GCD curves at current densities of 1–10 A/g, (**g**) calculated specific capacity values from GCD curves, (**h**) Nyquist plot of Co_3_O_4_ and the Co_3_O_4_@Mo-Co_3_O_4_ heterostructure_,_ (**i**) capacitance retention over 4000 GCD cycles; inset: ten consecutive GCD cycles.

**Figure 6:**
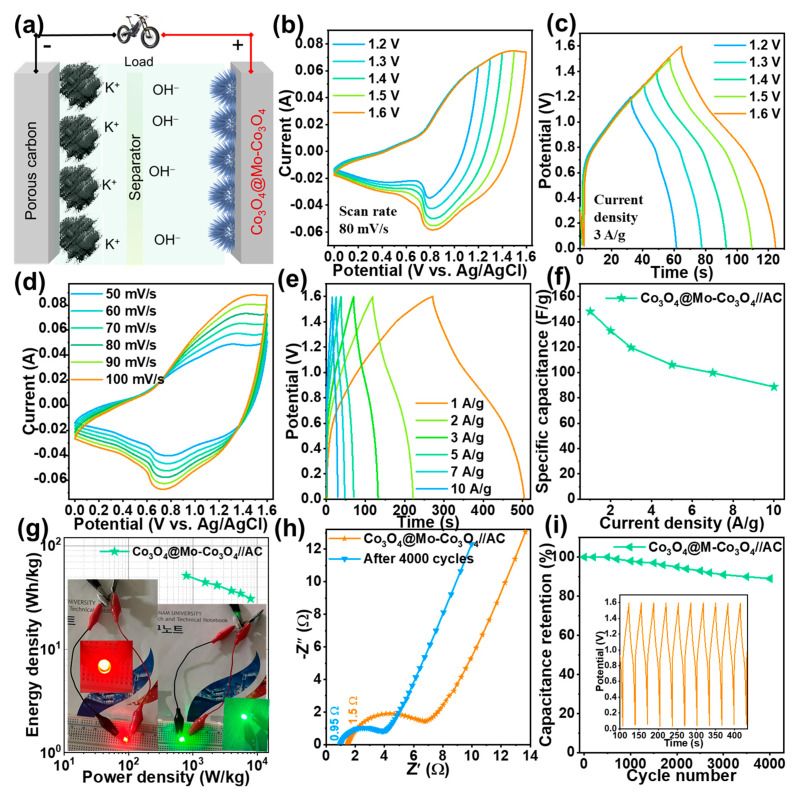
(**a**) Schematic of ASC device, (**b**) CV curves for different voltage windows at an 80-mV/s scan rate, (**c**) GCD curves for different voltage windows at a current density of 3 A/g (**d**,**e**) CV curves at scan rates of 50–100 mV/s and GCD curves at current densities of 1–10 A/g, (**f**) calculated specific capacitance values, (**g**) Power density vs. energy density plot, (**h**) Nyquist plot of before and after 4000 GCD cycles, (**i**) capacitance retention over 4000 GCD cycles; inset: ten consecutive GCD cycles of Co_3_O_4_@Mo-Co_3_O_4_//AC ASC device.

The hydrothermally obtained heterostructures were tested for three-electrode system electrochemical performance in 2M KOH electrolytes. Figure 5 illustrates a comparison of the Co_3_O_4_ and Co_3_O_4_@Mo-Co_3_O_4_ needle-like nanosheet heterostructure electrodes. It was found that the doping of Mo into Co_3_O_4_ enhanced the electrochemical properties, such as peak current and discharge time (Figure 5a,b). Notably, the cyclic voltammetry (CV) curves and the galvanic charge-discharge (GCD) curves demonstrated that doping did not shift the redox peaks of the Co_3_O_4_. This indicated that Co_3_O_4_ undergoes redox reactions while Mo facilitates the electrochemical performance of Co_3_O_4_. The CV curves in the potential window of −0.1 to 0.6 V at different scan rates, i.e., 10–30 mV/s, obtained for Co_3_O_4_ and the Co_3_O_4_@Mo-Co_3_O_4_ heterostructures, are shown in Figure 6c,d. At each scan rate, clear oxidation and reduction peaks (0.35 V and 0.2 V) were found in the same place, with an increase in the current density with increasing scan rate. Figure 5e,f presents the GCD profiles in a potential window of 0 to 0.45 V at different current densities, i.e., 1–10 A/g, obtained for Co_3_O_4_ and the Co_3_O_4_@Mo-Co_3_O_4_ heterostructure. The battery-type behavior is clearly visible in the plateau-like charge/discharge pattern. The Co_3_O_4_ and Co_3_O_4_@Mo-Co_3_O_4_ heterostructure revealed similar humps, indicating the occurrence of redox reactions due to cobalt, where Mo-Co_3_O_4_ has a higher discharge time than Co_3_O_4_. The Mo atoms did not show redox activity during charge/discharge reactions but promoted electrochemical performance. The calculated specific capacity is given in Figure 5g. The faradaic reactions for Co_3_O_4_ are given below [28]:

Co_3_O_4_ + H_2_O + OH^−^ ↔ 3CoOOH + e^−^ (6)

CoOOH + OH^−^ ↔ CoO_2_ + H_2_O + e^−^ (7)

The advantage of Mo-doping is to facilitate the electrochemical ability. The heterostructure formed by the addition of Mo improved the charge storage capacity by 30%. The pristine Co_3_O_4_ electrode exhibited a specific capacity of 615 C/g (1464 F/g), whereas the Co_3_O_4_@Mo-Co_3_O_4_ needle-like nanosheet heterostructure had a specific capacity of 814 C/g (1850 F/g). Besides Mo-doping, the enhanced electrochemical performance could be attributed to the heterostructure architecture of the obtained nanomaterials. The Mo-Co_3_O_4_ flower-like nanosheets provided a large area, leading to better electrolyte diffusion into the electrodes. In addition, the Mo-Co_3_O_4_ flower-like nanosheets provided an active electrochemical surface for redox reactions. The self-supported structure comprised a large surface area for charge storage and fast electron transfer reactions. The advantages of this morphology are reflected in the Nyquist plot given in Figure 5h. The solution resistance was found to be 1 Ω and 0.65 Ω for Co_3_O_4_ and for the Co_3_O_4_@Mo-Co_3_O_4_ heterostructure, respectively (inset in Figure 5h). However, the reduced resistance would have increased the charge kinetics during the electrochemical process. Further, the stability test for 4000 cycles of continuous charge/discharge process in the KOH electrolyte was done with the Co_3_O_4_@Mo-Co_3_O_4_ heterostructure electrode. Even at the 4000th cycle, around 90% capacity retention was observed for the Co_3_O_4_@Mo-Co_3_O_4_ electrode. The inset of the last 10 similar GCD cycles corroborates the structural stability of the Co_3_O_4_@Mo-Co_3_O_4_ heterostructure electrode during long-term cycling. Thus, the excellent morphology and crystallinity of the Co_3_O_4_@Mo-Co_3_O_4_ needle-like nanosheet heterostructure electrode indicate that it is a very promising electrode material for supercapacitor applications.

### 3.2. Electrochemical Properties of a Co_3_O_4_@Mo-Co_3_O_4_//AC Device

For practical applications, the Co_3_O_4_@Mo-Co_3_O_4_ heterostructure electrode was assembled with an AC electrode in a KOH electrolyte as an asymmetric supercapacitor, as represented in Figure 6a. The comparative CV curves of the negative AC and positive Co_3_O_4_@Mo-Co_3_O_4_ electrodes are presented in Figure S1. The asymmetric (Co_3_O_4_@Mo-Co_3_O_4_//AC) device potential window was optimized, as shown in Figure 6b,c. The CV curves in Figure 6b at 80 mV/s and the GCD profile at a current density of 3 A/g (Figure 6c) were made at different potential windows. The optimum potential window for the constructed device was found to be 0–1.6 V. The CV curves for the device were obtained at different scan rates, i.e., 50–100 mV/s, and GCD curves at various current densities, i.e., 1–10 A/g, as demonstrated in Figure 6d,e.

The calculated specific capacity for the Co_3_O_4_@Mo-Co_3_O_4_//AC device is shown in Figure 6f. It showed a maximum capacitance of 154.6 F g^−1^ at a current density of 1 A g^−1^. The superior supercapacitor performance might be accredited to the strong self-supported structure. The Ragone plot (Figure 6g) illustrated the energy and power density of the asymmetric Co_3_O_4_@Mo-Co_3_O_4_//AC device. The device exhibited an energy density of 51.35 Wh/kg at a power density of 790 W/kg. The values of Co_3_O_4_@Mo-Co_3_O_4_//AC device from the GCD are shown in Table 3, affirms improved capacitive performance of fabricated Co_3_O_4_@Mo-Co_3_O_4_//AC device compared to previously reported works. 

The EIS study summarized in Figure 6h reveals minimal increase in solution resistance, even after 4000 cycles of charge/discharge. As shown in Figure 6i, the high retention (89%) also proved the long-term durability of the asymmetric device. Thus, the Co_3_O_4_@Mo-Co_3_O_4_//AC device is promising for use in advanced electronics. Such significant electrochemical outcomes could be attributed to the crystallinity and morphology of the Co_3_O_4_@Mo-Co_3_O_4_ needle-like nanosheet heterostructure electrode, the self-supported structure with good mechanical and chemical stability, and the numerous nanoneedles, which offer enough surface area and active sites for electrochemical reactions and electrolyte intake. Additionally, the one-pot synthesis of the aforementioned heterostructure is a facile, rapid and cheap method.

## 4. Conclusions

A novel Co_3_O_4_@Mo-Co_3_O_4_ nanosheet composite for supercapacitor applications was synthesized and its physical/electrochemical characteristics were investigated. The Co_3_O_4_@Mo-Co_3_O_4_ nanosheet composite achieved a superior specific capacity of 814 C g^−1^ at 1 A g^−1^ and capacity retention of 90% with a good rate capability. The asymmetric SC fabricated using this composite material achieved a capacitance of 154.6 F g^−1^ at 1 A g^−1^, a specific energy of 51.35 Wh Kg^−1^, and a specific power of 790 W kg^−1^. Moreover, the Co_3_O_4_@Mo-Co_3_O_4_//AC device possesses superior rate capabilities and long cycles, with 89.7% of the starting capacitance remaining after 4000 continuous cycles at 2 A g^−1^. From this work, it may be concluded that the nanocomposite Co_3_O_4_@Mo-Co_3_O_4_ nanosheets coated over a Ni foam skeleton displayed superior capacities and could be considered for use in ultra-capacitor devices in the future.

## Figures and Tables

**Table 1:** Estimated lattice structure parameters and refinement parameter values from Rietveld refinement of Co_3_O_4_@Mo-Co_3_O_4_ heterostructure.

Unit Cell Parameters	
a, (Å)	8.08
b, (Å)	8.08
c, (Å)	8.08
α, (°)	90
β, (°)	90
γ, (°)	90
Cell volume (Å)^3^	528.24
Density (g/cm^3^)	6.05
Crystal system and Space group number	Cubic & (Fd-3m, 227)
**Structure parameters**	
Atoms	130
Bonds	168
Polyhedra	34
**Refinement parameters**	
R_p_	1.762
R_wp_	2.216
Goodness of fit (GOF)	0.805
R-Structure factor	4.68
R-Bragg factor	6.89

**Table 2:** Site occupancies of various ions along with their fractional coordinates.

Element	X	Y	Z	Occupancy	Site	Sym.
O1	0.14000	0.14000	0.14000	1.000	16e	0.3 m
O2	0.61000	0.61000	0.61000	1.000	16e	0.3 m
Co1	0.75000	0.75000	0.75000	1.000	4d	−43 m
Co2	0.00000	0.00000	0.00000	1.000	4a	−43 m
Co3	0.37500	0.37500	0.37500	1.000	16e	0.3 m

**Table 3:** Various electrochemical properties of Co_3_O_4_-related materials compared to previous reports.

Electrode	Electrolyte	Specific Capacitance	Retention Rate	Energy Density (Wh kg^−1^)	Power Density (W kg^−1^)	Ref.
Mn@Co_3_O_4_	2M KOH	773 F g^−1^	73.9%/5000 cycles	NA	NA	[29]
Mn-Co_3_O_4_/NF	2M KOH	668.4 F g^−1^	104%/10,000	10.63	14,700	[30]
V-Co_3_O_4_	3M KOH	1593 F g^−1^	NA	66.88	240	[31]
Fe-Co_3_O_4_	6M KOH	767.9 C g^−1^	90%/4000	37	750	[32]
Cr, Sn- Co_3_O_4_	3M KOH	1413.56 Fg^−1^	89.41%/3000	NA	NA	[33]
Ce-Co_3_O_4_	3M KOH	1309.6 F g^−1^	90.86%/2000	NA	NA	[34]
Mo-Co3O4	2M KOH	1850 F g^−1^	90%/4000	--	--	This Work
Mo-Co3O4//AC	2M KOH	148 F g^−1^	89%/4000	51.4	790	

## Data Availability

No new data were created or analyzed in this study. Data sharing is not applicable to this article.

## Supplementary Materials

The following supporting information can be downloaded at: https://www.mdpi.com/article/10.3390/nano12142330/s1. Figure S1. The Rietveld refinement pattern of Co_3_O_4_@Mo-Co_3_O_4_ heterostructure; Figure S2. FTIR spectra of Co_3_O_4_ and Co_3_O_4_@Mo-Co_3_O_4_ composite; Figure S3. CV curves of two electrodes positive and negative.
