# Supplementary material for: Self-Supported Co3O4@Mo-Co3O4 Needle-like Nanosheet Heterostructured Architectures of Battery-Type Electrodes for High-Performance Asymmetric Supercapacitors
Source: Nanomaterials (Basel). 2022 Jul 7;12(14):2330. doi: 10.3390/nano12142330 (PMC9324492; doi:10.3390/nano12142330)
Supplement: Supplementary file 1 [file nanomaterials-12-02330-s001.zip › nanomaterials-1806251-supplementary.pdf]

# Supplementary Materials

# Self-Supported $Co_3O_4$@Mo-$Co_3O_4$ Needle-Like Nanosheet Heterostructured Architectures of Battery-Type Electrodes for High-Performance Asymmetric Supercapacitors

**Yedluri Anil Kumar [1,2,†], Himadri Tanaya Das [3], Phaneendra Reddy Guddeti [4], Ramesh Reddy Nallapureddy [5,†], Mohan Reddy Pallavolu [5,*], Salem Alzahmi [6,2] and Ihab M. Obaidat [1,2,*]**

[1] Department of Physics, United Arab Emirates University, Al Ain 15551, United Arab Emirates; yedluri.anil@gmail.com
[2] National Water and Energy Center, United Arab Emirates University, Al Ain P.O. Box 15551, United Arab Emirates
[3] Centre of Advanced Materials and Applications, Utkal University, Vanivihar, Bhubaneswar 751004, India; himadridas@utkaluniversity.ac.in
[4] Department of Physics, Sri Venkateswara Vedic University, Tirupati 517502, India; phaneendra369@gmail.com
[5] School of Chemical Engineering, Yeungnam University, Gyeongsan 38541, Korea; rameshsun999@gmail.com
[6] Department of Chemical & Petroleum Engineering, United Arab Emirates University, Al Ain 15551, United Arab Emirates; s.alzahmi@uaeu.ac.ae
* Correspondence: pmreddy@yu.ac.kr (M.R.P.); iobaidat@uaeu.ac.ae (I.M.O.)
† These authors contributed equally to this work.

## 1. Characterization details

The Transmission electron microscope (TEM; Tecnai G$^2$ F20 STwin) was used for recording the microstructure of electrode material. A field emission scanning electron microscope (FESEM; Hitachi S-4800) was used for surface analysis and energy dispersive X-ray spectroscopy (EDS) for compositional analysis. X-ray diffraction (XRD; MPD for bulk, 3 kW, PAN analytical X'Pert PRO) was used to measure the diffraction patterns with Cu K$\alpha$ radiations ($\lambda$ = 1.54056 Å). The chemical composition and oxidation states was determined by the X-ray photoelectron spectroscopy (XPS; K-alpha, Thermo Scientific, USA) with Al K$\alpha$ radiation. Raman spectroscopy (Horiba Jobin Yvon HR 800 UV) with Nd: YAG laser source ($\lambda$ = 532 nm) was used to further examie the structural properties. The Fourier Transform Infrared Spectroscopy (FTIR) (model5300 Jasco, USA) was used to find the bonding information in the wavelength range of 400 to 4000 cm$^{-1}$.

Using the electrochemical workstation of CHI 760E, the electrochemical characteristics of $Co_3O_4$ and Mo-$Co_3O_4$ composites were studied in two and three electrode arrangements. The supercapacir performance of the electrodes was investigated using several electrochemical properties like galvanostatic charge/discharge (GCD), cyclic voltammetry (CV), and electrochemical impedance spectroscopy (EIS). The working electrode was made-up by brush coating. The slurry was composed of an 80:10:10 ratio of active materials ($Co_3O_4$ and Mo-$Co_3O_4$), conductive carbons, and polyvinylidene fluorides (PVDF). The slurry was mixed homogeneously with NMP and the slurry was brush-coated on a pre-cleaned Ni-foam. These Ni-foams were dried at 90 $^{o}$C/8h in an oven to remove the additive mixtures.

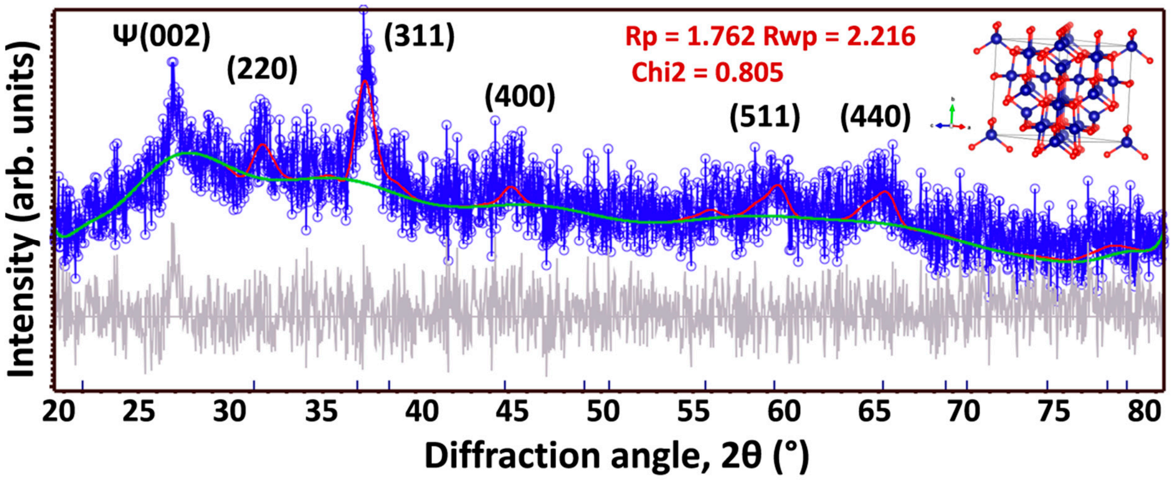


**Figure S1.** The Rietveld refinement pattern of $Co_3O_4$@Mo-$Co_3O_4$ heterostructure.

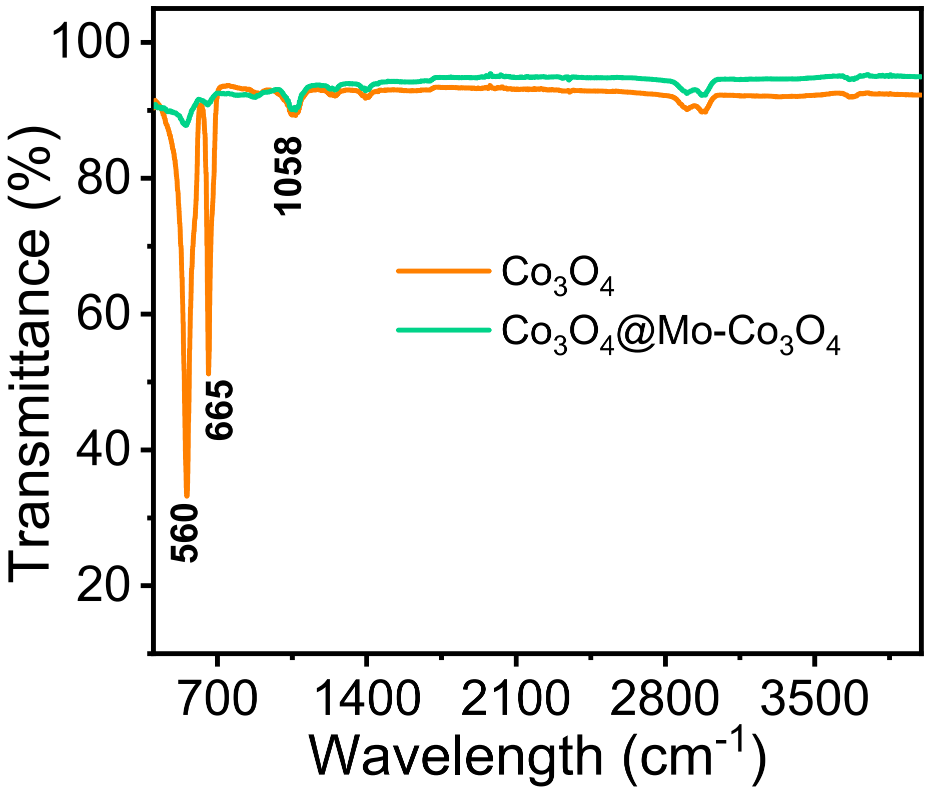


**Figure S2.** FTIR spectra of Co3O4 and $Co_3O_4$@Mo-$Co_3O_4$ composite.

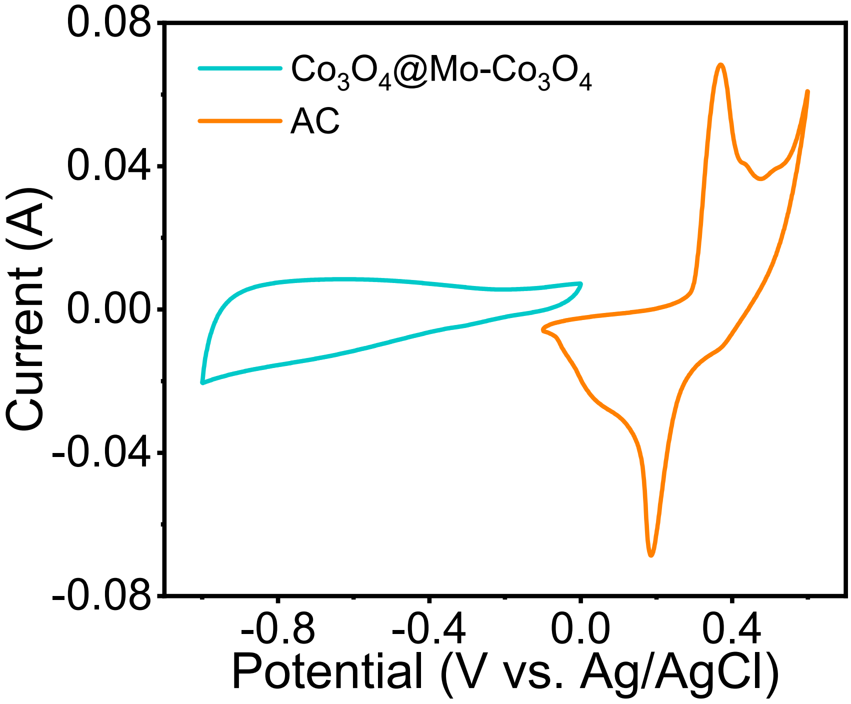


**Figure S3.** CV curves of two electrodes positive and negative
